# Seeking a geochemical identifier for authigenic carbonate

**DOI:** 10.1038/ncomms10885

**Published:** 2016-03-07

**Authors:** Ming-Yu Zhao, Yong-Fei Zheng, Yan-Yan Zhao

**Affiliations:** 1 CAS Key Laboratory of Crust-Mantle Materials and Environments, School of Earth and Space Sciences, University of Science and Technology of China, Hefei 230026, China

## Abstract

Authigenic carbonate was recently invoked as a third major global carbon sink in addition to primary marine carbonate and organic carbon. Distinguishing the two carbonate sinks is fundamental to our understanding of Earth's carbon cycle and its role in regulating the evolution of atmospheric oxygen. Here, using microscale geochemical measurements of carbonates in Early Triassic strata, we show that the growth of authigenic carbonate follows a different trajectory from primary marine carbonate in a cross-plot of uranium concentration and carbon isotope composition. Thus, a combination of the two geochemical variables is able to distinguish between the two carbonate sinks. The temporal distribution of authigenic carbonates in the Early Triassic strata suggests that the increase in the extent of carbonate authigenesis acted as a negative feedback to the elevated atmospheric CO_2_ concentration.

## Abstract (web summary)

Distinguishing between authigenic carbonate and primary marine carbonate is fundamental to our understanding of Earth's carbon, oxygen and calcium cycles. Here, the authors show that a combination of uranium concentration and carbon isotope composition is able to distinguish between the two carbonate sinks.

## Graphical abstract

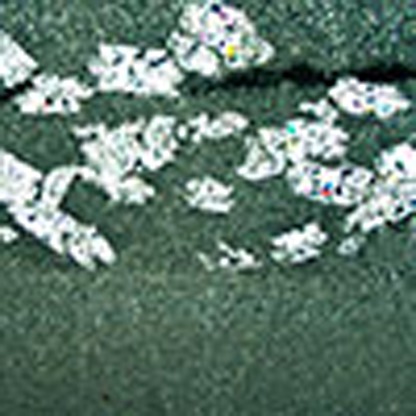

The global carbon cycle shapes the biosphere and atmosphere, and regulates the redox and heat budgets of our planet. However, our understanding of the basic framework of this biogeochemical cycle is still very rudimentary. Traditionally, carbon entering the Earth's surficial spheres is hypothetically removed as two major sinks, namely primary marine carbonate and organic carbon^1^,^2^. Recently, however, it was suggested that authigenic carbonate is a considerable component of modern oceanic carbon sequestration^3^ and probably a more essential carbon sink during periods with prevalent oceanic oxygen deficiency in Earth history^4^. Quantitative recognition of the status of authigenic carbonate in global carbon, oxygen and calcium cycles^3^,^4^,^5^ requires a clear way to distinguish it from primary marine carbonate, which may also be important in the interpretation of carbon isotope shifts recorded in marine carbonates.

Authigenic carbonates, including calcite, dolomite and siderite, can be precipitated in anoxic pore fluids, in response to elevated alkalinity resulting from a series of processes involving organic-matter degradation, such as iron reduction, sulphate reduction, methanogenesis and thermocatalytic decarboxylation^6^,^7^,^8^. With carbon derived partially from these processes, authigenic carbonates can have distinct carbon isotopic compositions from those of primary marine carbonates, which makes them important in our understanding of Earth's coupled carbon and oxygen cycles^4^,^5^. Most known authigenic carbonates associated with anoxic organic-matter degradation were likely formed in the sulphate reduction zone and the sulphate–methane transition zone^7^, although some authigenic dolomite can be precipitated deeper in the zone of methanogenesis to thermal decarboxylation^6^,^9^,^10^.

Authigenic carbonates can occur in association with fluid venting at methane seeps, which is sporadically distributed over the modern seafloor^11^ and may be enhanced by a drop in deep seawater oxygen level^12^. The more widespread type of authigenic carbonate, however, probably exists in ordinary sediments without methane seep structures. Identifying this authigenic carbonate sink in deep time presents a unique challenge because it may be morphologically and isotopically indistinguishable from primary marine carbonate, it is possibly mixed with primary marine carbonate at the microscale^4^, and the existing modern analogues^13^,^14^ are imperfect.

Here we present detailed petrographic and geochemical observations of Early Triassic strata. The results show that the uranium concentration combined with the carbon isotope composition can be used to distinguish Earth's two carbonate sinks.

## Results

### Stratigraphy

**Figure 1:**
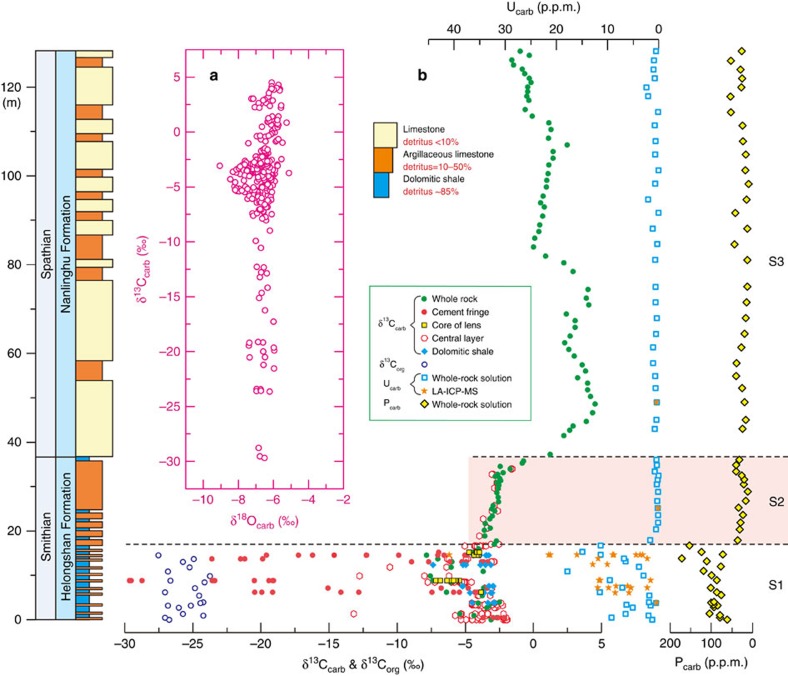
Geochemical results of sedimentary carbonates from the Yindushan section. (**a**) A δ^13^C_carb_–δ^18^O_carb_ cross-plot for all samples. (**b**) Stratigraphic variations of δ^13^C_carb_, δ^13^C_org_, P_carb_ and U_carb_. S1, S2 and S3 represent three stratigraphic intervals divided by δ^13^C_carb_ values.

We sampled a stratigraphic section spanning from the middle Smithian to the late Spathian at a high resolution in the deep-water zone of the Lower Yangtze basin, South China (Supplementary Note 1 and Supplementary Fig. 1). This mixed carbonate and siliciclastic sequence contains three lithologically distinguishable units (Fig. 1) comprising a basal sequence (S1) dominated by dolomitic shale, intercalated by thin layers (2–10 cm) of argillaceous limestone or oblate limestone lenses (with thicknesses of 2–5 cm and diameters >1 m); a middle sequence (S2) characterized by argillaceous limestone, interbedded by a few thin layers of dolomitic shale; and an upper sequence (S3) containing limestone and argillaceous limestone.

### Macroscale geochemistry

Carbonate carbon isotope values (δ^13^C_carb_) of whole-rock samples of the limestone lenses/layers in S1 show point-to-point differences between successive stratigraphic samples and can be lower than the mantle δ^13^C value of −5‰ (Fig. 1 and Supplementary Table 1). In contrast, whole-rock samples of limestone and argillaceous limestone layers in the subsequent S2 and S3 show point-to-point coherent δ^13^C_carb_ values >−4‰. Carbonate phosphorus and uranium concentrations (P_carb_ and U_carb_) of whole-rock samples of S1 are elevated relative to those of S2 and S3 (Fig. 1 and Supplementary Table 2). P_carb_ and U_carb_ of whole-rock samples exhibit no obvious correlation with aluminium (*r*=0.17 and 0.02, respectively), reflecting minor contamination from silicates.

The organic carbon and sulphur concentration data of HCl-insoluble residues from the three intervals mainly fall above the mean correlation line of normal marine sediments^15^ (Supplementary Fig. 2), indicating euxinic conditions throughout the entire profile.

### Microscale petrography and geochemistry

**Figure 2:**
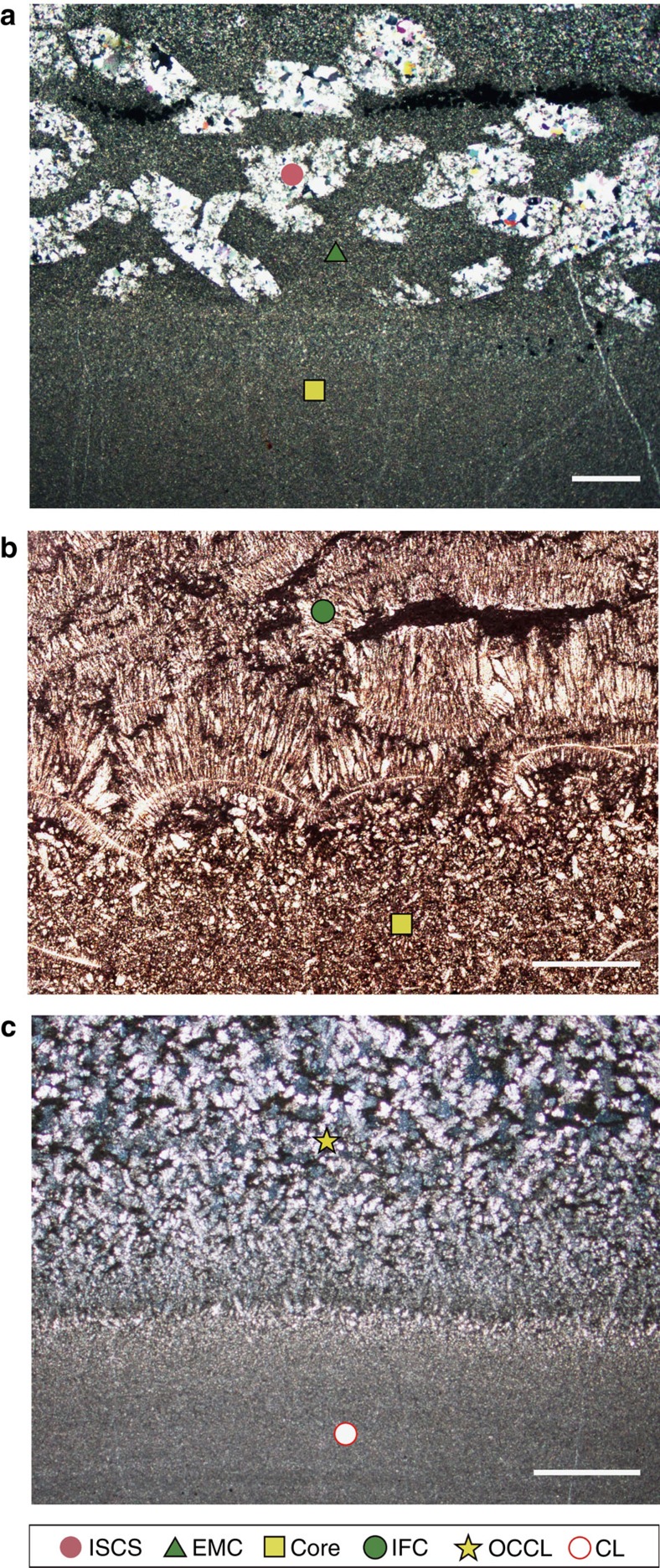
Textures of the limestone lenses/layers in S1. (**a**) A limestone lens core surrounded by a cement fringe consisting of equigranular microcrystalline calcite (EMC), irregular-stacked calcite spar (ISCS) and pyrite bands (black). (**b**) A limestone lens core surrounded by layers of isopachous fibrous calcite (IFC). (**c**) The central layer (CL) of a thin limestone layer surrounded by outward-coarsening calcite laminae (OCCL). δ^13^C_carb_ values of subsamples are shown in Fig. 3. Scale bar, 1 mm. **a** and **c** are shown with cross-polarized light, whereas **b** is shown with plane-polarized light.

We performed a detailed investigation of S1 of the Early Triassic section. The thin limestone lenses/layers of S1 have a core/central layer containing homogeneous microcrystalline calcite (Fig. 2 and Supplementary Fig. 3) and dispersed xenomorphic or framboidal pyrite (5–60 μm, Supplementary Fig. 4), surrounded or not by a cement fringe (with a thickness of 0.5–10 mm).

**Figure 3:**
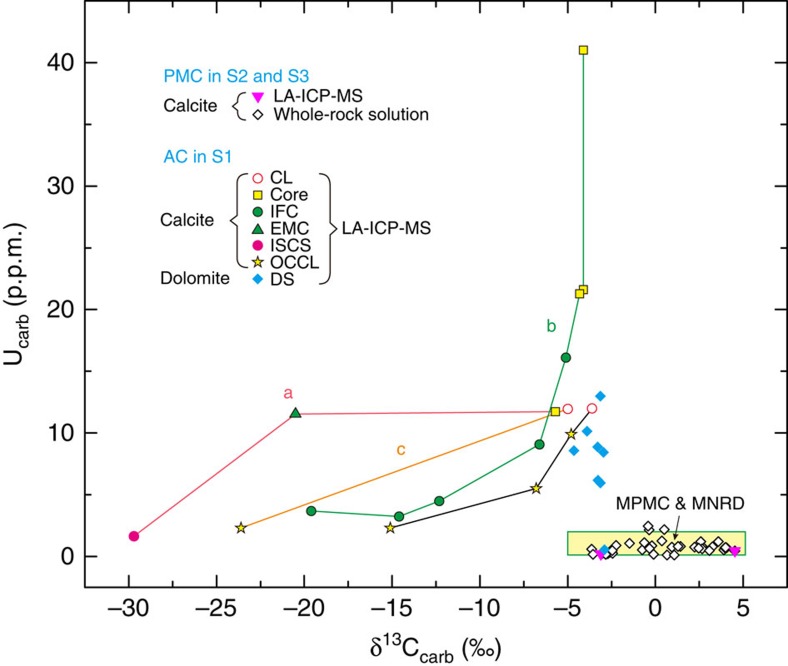
A cross-plot of δ^13^C_carb_ and U_carb_ data for the Yindushan section. Lines a, b and c correspond to the three samples shown in Fig. 2. The direction of the authigenic calcite growth sequence, generally from the centre of the core/central layer to the outside edge of the cement fringe, is from right to left for each line. S1, S2 and S3 represent three stratigraphic intervals shown in Fig. 1. AC, authigenic carbonates; CL, central layer; DS, dolomitic shale; EMC, equigranular microcrystalline calcite; IFC, isopachous fibrous calcite; ISCS, irregular-stacked calcite spar; MNRD, modern non-skeletal replacement dolomites; MPMC, modern primary marine calcites; OCCL, outward-coarsening calcite laminae; PMC, primary marine carbonates.

The cement fringes in S1 consistently surround the oblate cores (with diameters >1 m) and the central layers, suggesting that they grew *in situ* on the cores/central layers. The investigated cement fringes can be categorized into two types according to their growth modes. The first type is composed of equigranular microcrystalline calcite, irregular-stacked calcite spar and bands (with a width of 0.2–2 mm) of pyrite (Fig. 2a and Supplementary Figs 3 and 4). δ^13^C_carb_ values are distinct between the equigranular microcrystalline calcite and the irregular-stacked calcite spar, but are homogeneous within each of these two calcite groups (Fig. 3 and Supplementary Table 1). The precipitation of substantial pyrite is likely to have stimulated a transient high level of carbonate oversaturation^16^, rendering large-scale nucleation. Thus, we speculate a pervasive growth mode^16^,^17^ for this cement type. The second type, which shows an outward growth trend relative to the cores/central layers, consists of layers of isopachous fibrous calcite (Fig. 2b) or outward-coarsening calcite laminae (Fig. 2c), with a pyrite distribution similar to those of the cores/central layers (Supplementary Fig. 4). This cement type can also exhibit outward variations in geochemical composition relative to the cores/central layers (Fig. 3) and thus is likely to have a concentric growth mode^17^.

A remarkable variation of δ^13^C_carb_ from −29.7 to −2.2‰ occurs in the cement fringes of S1 (Figs 1 and 3). The lowest δ^13^C_carb_ value of −29.7‰ occurring in the irregular-stacked calcite spar is even lower than the concomitant δ^13^C_org_ value of −26.6‰. Although the lowest δ^13^C_carb_ value may not necessarily be caused by the involvement of methane, the possibility of methane oxidation cannot be ruled out.

The δ^13^C_carb_ values of the cores/central layers in S1 range from −13.2 to −1.9‰. Microscale analyses of δ^13^C_carb_ and U_carb_ reveal obvious decreasing trends from the cores/central layers to the cement fringes (Fig. 3 and Supplementary Table 3), with U_carb_ decreasing markedly from 41.0–1.7 p.p.m. Although the δ^13^C_carb_ values and mineral morphology of the cores/central layers of S1 are often indistinguishable from those of primary marine carbonate, their U_carb_ concentrations are considerably higher.

The ferroan dolomite (with ∼2.4 mol% iron and ∼1,560 p.p.m. manganese) in the dolomitic shale exists as scattered rhombs (Supplementary Fig. 5), with diameters mostly in the range of 20–30 μm and some up to 50 μm. As revealed by backscattered electron images (Supplementary Fig. 5), the ferroan dolomite grains usually have an iron-poor core surrounded by an iron-rich rim. The dolomitic shale samples have δ^13^C_carb_ values of −7.4 to −2.9‰ (Fig. 1), overlapping with those of primary marine carbonate, but most dolomitic shale samples have higher U_carb_ concentrations, in the range of 5.9–13 p.p.m. (Fig. 3).

Regardless of the large magnitude of the δ^13^C_carb_ variation, the δ^18^O_carb_ values of the entire profile are within a narrow range of −9.0 to −5.1‰ (Fig. 1).

## Discussion

Uranium is a water-soluble element, and thus its concentration in carbonate records the composition of the fluid from which the carbonate was precipitated^18^. We interpret the elevation of the carbonate uranium concentrations of S1 relative to those of S2 and S3 (Fig. 1) as resulting from different valences and carbonate-water partition coefficients of uranium between anoxic pore fluid and seawater. In oxic and even anoxic seawater, dissolved uranium exists predominantly as U(VI) in the form of uranyl and uranyl carbonate ions^19^,^20^,^21^ and is conservative with a concentration of ∼13 nmol kg^−1^ and a residence time of ∼0.5 Myr. Likely because of the much larger size of uranyl ion relative to Ca(II), the partition coefficient of U(VI) species in carbonate is low and is in the range of 0.007–0.2 for calcite^21^,^22^,^23^. However, in anoxic sediments, uranium may be taken up by carbonate in the form of U(IV)^24^, which is likely to have a much higher partition coefficient because the ionic radius of U(IV) (0.89 Å) is similar to that of Ca(II) (0.99 Å). In anoxic seawater, the reduction of U(VI) to U(IV) is thermodynamically favoured but kinetically hindered, possibly due to the requirement for mineral surfaces in the reduction of uranium^19^,^25^. Thus, the reduction and removal of dissolved uranium principally occurs in the zones of iron and sulphate reduction in suboxic and anoxic sediments^26^,^27^,^28^, tightly coupled with the formation of early-stage authigenic carbonate.

**Figure 4:**
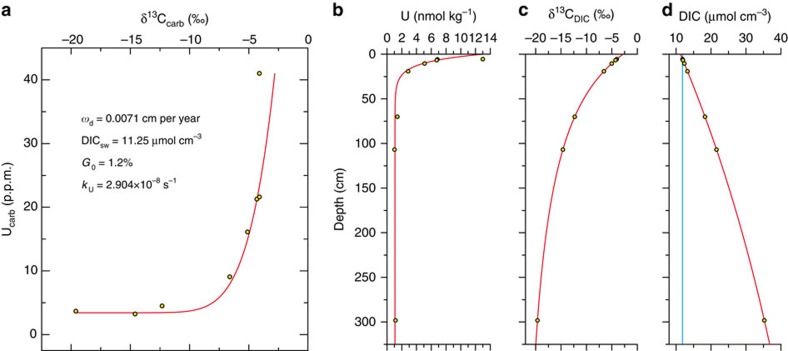
Modelled variations of authigenic carbonate compositions and chemical profiles of pore water. The sample in the model corresponds to line b in Fig. 3. (**a**) A modelled co-variation of δ^13^C_carb_ and U_carb_. (**b**–**d**) Modelled pore water profiles of uranium and DIC concentrations and δ^13^C_DIC_. The blue line in **d** shows the DIC concentration at which the authigenic calcite started to grow. The calcite-pore water partition coefficient of uranium is 13.25, with the assumption that the uranium and calcium concentrations of the pore water corresponding to the earliest authigenic calcite are the same as those of modern ocean water (13 nmol kg^−1^ and 0.01 mol kg^−1^, respectively). See Supplementary Table 6 for the values/ranges of all parameters in the model.

The outward variations of δ^13^C_carb_ and U_carb_ in S1 (Fig. 3) represent geochemical variations in the pore water profile across which the limestone layers/lenses gradually grew. On the modern seafloor, the uranium concentration of pore waters is typically observed to gradually decrease from the iron reduction zone to the sulphate reduction zone (below the iron maximum) and remain constant at depth^26^,^27^,^29^. A typical δ^13^C profile of dissolved inorganic carbon (DIC) in pore water shows an initial decrease from a value close to that of the bottom seawater (∼0‰) in the iron reduction zone to approximately −25‰ near the base of the sulphate reduction zone, then an increase to approximately +15‰ in the methanogenic zone, and finally a decrease to approximately −10‰ near the base of the methanogenic zone within the thermal decarboxylation zone^6^,^7^,^8^,^9^,^10^. We use two diagenetic equations to simulate the outward variations in chemical compositions of authigenic calcite and the corresponding pore water chemistry (Fig. 4 and Supplementary Fig. 6). The model reproduces the observed correlation between δ^13^C_carb_ and U_carb_, suggesting that the growth of authigenic calcite is likely to have started at a position near the top of the sulphate reduction zone and ended at the bottom of this zone.

Primary marine carbonates normally have δ^13^C values higher than the mantle value of −5‰. Modern primary marine calcites^30^ and modern non-skeletal replacement dolomites formed through dolomitization^31^ commonly have uranium concentrations <1 and 2 p.p.m., respectively. Our limited analyses of the non-authigenic microcrystalline dolomites from Triassic and Ediacaran times also yield uranium concentrations of <2 p.p.m. (Supplementary Tables 4 and 5). As shown in Fig. 3, early-stage authigenic calcite has much higher uranium concentrations than those of primary marine calcites, whereas late-stage authigenic calcite has much lower δ^13^C_carb_ values. The uranium concentrations of the late-stage authigenic calcite (>1.7 p.p.m.) are also generally above or at the top end of the range of primary marine calcites (<2 p.p.m.), likely resulting from a stable and considerable total uranium concentration of pore water at depth^26^,^29^,^32^. Although the degree of uranium enrichment in authigenic carbonate may vary as a function of the seawater uranium concentrations in Earth history, the relative enrichment of uranium between authigenic carbonates and contemporaneous primary marine carbonates likely holds given the geochemical behaviours of uranium outlined above. δ^13^C_carb_ alone is not sufficient to distinguish authigenic carbonates, due to the overlap in the δ^13^C_carb_ ranges of the two carbonate sinks. Nevertheless, uranium concentrations combined with δ^13^C_carb_ can be used to differentiate the authigenic carbonate sink from the primary marine carbonate sink, and *in situ* measurements allow authigenic carbonate to be identified at the microscale.

Given the deep-water sedimentary environment, the large sizes and the considerable iron and manganese concentrations, the ferroan dolomite of the dolomitic shale in S1 was formed within the sediments. Dissolved iron is usually depleted in the sulphate reduction zone, due to the formation of pyrite, but its enrichment has been frequently observed in the methanogenic zone^33^. Accordingly, it has been revealed that sulphate-reduction dolomites are generally deficient in iron and manganese (<1 mol% and 300–400 p.p.m., respectively) relative to methanogenetic dolomites (>2 mol% and 1,200–1,400 p.p.m., respectively)^8^,^34^. Thus, in light of the high iron and manganese contents (∼2.4 mol% and ∼1,560 p.p.m., respectively) and core-rim structure (Supplementary Fig. 5), the ferroan dolomite in S1 is likely to have formed in the upper part of the methanogenic zone, where the iron concentration gradually increases^33^. This suggests that the method of identifying authigenic carbonates may also be applicable to the authigenic carbonate formed below the sulphate reduction zone because the uranium concentrations of the ferroan dolomite generally exceed those of non-skeletal replacement dolomites (Fig. 3). Similarly, a previous study revealed that Cambrian calcite concretions formed within and below the sulphate reduction zone also have high uranium concentrations^35^. Taken together, although more verifications are needed, the method of identifying authigenic carbonates is likely applicable to various diagenetic zones, including the iron reduction zone, sulphate reduction zone and methanogenic zone.

Authigenic carbonate is the dominant carbonate component in S1 because the limestone layers/lenses and the ferroan dolomite all have an authigenic origin. Authigenic carbonate also exists as ferroan dolomite in the shale of S2, but, as indicated by the U_carb_ and δ^13^C_carb_ results (Figs 1 and 3), the remaining limestone and argillaceous limestone layers of S2 and S3 are primary marine carbonates. The Smithian is known as a period with a marked global negative δ^13^C_carb_ shift^36^. It is likely that this carbon isotope shift was partially a reflection of a change in seawater chemistry because the primary marine calcite in S2 also has low δ^13^C_carb_ values (approximately −3‰). In view of its distribution in S1 and S2 (Fig. 1), the active carbonate authigenesis is likely to have been stimulated by the environmental forces accompanying the global negative carbon isotope shift. In addition to atmospheric CO_2_ and the oceanic oxygen level^37^, we propose that the influence of temperature on carbonate authigenesis may also be important because high temperature will enhance chemical weathering, and thus the generation rates of iron oxides^38^, which promote carbonate authigenesis^3^,^16^. Therefore, coupled global environmental forces in the Smithian^39^,^40^, such as high temperature, the spread of anoxia and the increased level of atmospheric CO_2_, may have collectively boosted laterally extensive carbonate authigenesis. Because severe silicate diagenesis is also likely associated with anoxia^41^, the active carbonate authigenesis along with severe silicate diagenesis may represent an additional negative feedback that stabilizes atmospheric CO_2_ concentrations.

Authigenic carbonate does not necessarily capture seawater conditions and thus should be identified before using carbonate records to reconstruct the compositions of ancient seawater. For example, in the Ediacaran Doushantuo Formation of the Yangtze platform, δ^13^C_carb_ results of dolomite layers in the black shale above the cap dolomite have been interpreted as a reflection of seawater compositions^42^. Our δ^13^C_carb_ and U_carb_ analyses show that the cap dolomite falls into the region of modern non-skeletal replacement dolomites (Supplementary Fig. 7 and Supplementary Table 4). However, with much higher U_carb_ concentrations (∼6 p.p.m.), the dolomite layers in the black shale fall into the region of Early Triassic authigenic carbonates, indicating their authigenic origin within the sediments.

The method of identifying authigenic carbonates allows us to investigate the influence of carbonate authigenesis on extreme carbon isotope events in deep time. We take the Ediacaran Shuram anomaly as an example because it is the largest known δ^13^C_carb_ shift in Earth history^43^ and has been linked to the rise of macroscopic bilaterians and calcifying metazoans^43^,^44^,^45^. The large magnitude (>10‰) and the lack of a simultaneous decrease in δ^13^C_org_ suggest that this δ^13^C_carb_ negative shift event may have been caused by the addition of authigenic carbonates to primary marine carbonates^4^. We investigated the third negative δ^13^C_carb_ excursion (N3) in the Ediacaran Doushantuo Formation of the Yangtze platform (Supplementary Note 1 and Supplementary Fig. 7), an interval corresponding to the Shuram anomaly^44^. The organic-rich shale (organic carbon contents >1%) below and within N3 has high uranium concentrations (14–90 p.p.m.) comparable to modern values^46^, indicating high oceanic uranium concentrations in this period. However, all the calcite and dolomite samples of N3 have uranium concentrations of <1.0 p.p.m. (Supplementary Fig. 7 and Supplementary Table 5), similar to the values of the underlying primary marine carbonates but much lower than those of the authigenic dolomite in the Ediacaran. Thus, at least in the investigated region, the Shuram anomaly is not likely to be an artefact of the incorporation of authigenic carbonate.

## Methods

### Sample processing

All the samples were devoid of visible alteration under a microscope. The carbonate phases of all the samples were identified using a Raman spectroscope, X-ray diffraction or the energy-spectrum-scanning function of scanning electron microscopy. The carbonate phase of limestone lenses and limestone layers in the entire profile was identified as calcite embedded by very sparse dolomite grains (Supplementary Fig. 3), whereas the carbonate phases of dolomitic shale are ferroan dolomite and calcite. Samples prepared for whole-rock analyses were broken into small chips to remove the veins before pulverization. After petrographic observation, microzone powders (80–200 μg) were obtained from polished slices using the high-speed drill of a Leica Micromill.

### δ^13^C_carb_ and δ^18^O_carb_ analyses

To analyse the carbon and oxygen isotopes in carbonate, 80–200 μg of powder was reacted with anhydrous phosphoric acid at 72 °C for ∼2 h through a GasBench II device connected to a Thermo Fisher Scientific MAT 253 mass spectrometer at the CAS Key Laboratory of Crust-Mantle Materials and Environments of the University of Science and Technology of China (USTC), Hefei. On the basis of duplicate analyses of a standard NBS-19, the external precisions (1*σ*) of the δ^13^C_carb_ and δ^18^O_carb_ analyses were better than ±0.1‰ and ±0.2‰, respectively. All δ^13^C_carb_ and δ^18^O_carb_ results are presented relative to Vienna Pee Dee Belemnite (VPDB). The detailed procedures for carbon and oxygen isotope analyses can be found in ref. 47.

### δ^13^C_org_ analysis

Preparation for organic carbon isotope analysis involved dissolution in 6 N hydrochloric acid to remove the carbonate, stepwise centrifugation to remove the acid and drying at 75 °C. The organic carbon isotopes were analysed on a DELTA plus XP+Conflo III+Flash EA, with a reaction tube at 1,020 °C, a reduction tube at 650 °C, a chromatographic column at 400 °C and a carrier gas flow of 90 ml min^−1^. On the basis of repeated analyses of a standard GBW04407, the external precision (1*σ*) was better than ±0.1‰. All δ^13^C_org_ data are presented relative to VPDB.

### Whole-rock major and trace element analyses

For the analyses of U_carb_, P_carb_ and Al concentrations in whole rocks, ∼50 mg of the powdered samples were cleaned in ultra-pure water, and then dissolved by ultra-pure 0.5 N acetic acid. The insoluble residues were removed through centrifugation; the resultant solutions were subsequently transformed into HNO_3_ and then spiked and diluted for inductively coupled plasma atomic emission spectrometry (ICP-AES) and ICP-mass spectrometry (ICP-MS) analyses. The detailed procedures can be found in ref. 18. The external precisions (1*σ*) of the U_carb_, P_carb_ and Al analyses were better than ±2%.

### Microscale U_carb_ analysis

Microdomain carbonate uranium content was determined by laser ablation (LA)-ICP-MS at the CAS Key Laboratory of Crust-Mantle Materials and Environments at USTC, using an ArF excimer ultraviolet (193 nm) system connected to an Agilent 7700 series ICP-MS. The analyses were conducted with a pulse rate of 10 Hz. The spot diameters were in the range of 16–60 μm, depending on the sizes of the calcite or dolomite grains. NIST 610 was used as the standard. To verify the validity of this method, two samples of primary marine carbonate (11CH738 and 11CH796) were analysed by both LA-ICP-MS and solution methods. The results of the LA-ICP-MS method (0.21 and 0.49 p.p.m., Fig. 3 and Supplementary Table 3) are comparable with those of the solution method (0.25 and 0.46 p.p.m., Supplementary Table 2).

### OC_IR_ and S_IR_ analyses

For the analyses of organic carbon and sulphur contents in detrital sediments, pretreatment procedures were the same as those used for the organic carbon isotope analysis. The organic carbon contents of insoluble residues were measured using an Elementar Vario EL tube, with a combustion tube at 950 °C and a reduction tube at 550 °C, and the sulphur contents of the insoluble residues were measured on an Elementar Vario EL III, with a combustion tube at 1,150 °C and a reduction tube at 850 °C. Uncertainties (1*σ*) in the analyses of organic carbon and sulphur contents were lower than ±0.3% and ±0.5%, respectively.

### Element mapping analysis

X-ray element mapping of samples was performed on a Tescan Mira 3 LMH FE-SEM at the CAS Key Laboratory of Crust-Mantle Materials and Environments at USTC, with a beam current of 20 nA and an acceleration voltage of 15.0 kV. The maps were generated with a dwell time of 200 ms per pixel and at a resolution of 512 × 400 pixels. The area per pixel is ∼0.264 μm × 0.264 μm.

### Numerical modelling

We use the following one-dimensional numerical model to describe the uranium concentration profile of pore water:


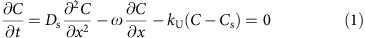


where *C* is the concentration of dissolved uranium, *x* is the depth (with the downward direction taken to be positive), *D*_s_ is the sediment diffusion coefficient, *ω* is the sediment advection rate, *k*_U_ is the first-order removal rate constant of uranium and *C*_s_ is the stable uranium concentration at depth. *D*_s_*=D*_M_* × φ*^*2*^, where *D*_M_ is the free solution diffusion coefficient and *φ* is the porosity. *ω=ω*_d_/(1−*φ*), where *ω*_d_ is the sedimentation rate of compacted Smithian strata in the study area. *k*_U_ is used because the removal rate of uranium is first order with respect to the uranium concentration^27^. We introduce the *C*_s_ term because the uranium concentration of pore water is usually relatively stable and considerable (1.6–9.9 nmol kg^−1^) at depth^26^,^29^,^32^. The boundary conditions of equation (1) are as follows:


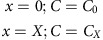


where *C*_0_ is the dissolved uranium concentration of seawater and *C*_*X*_ is the dissolved uranium concentration at depth *X*. Solving equation (1) for the boundary conditions yields:


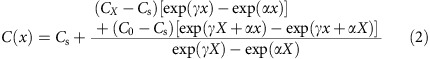


where


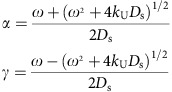


If the value of *X* is sufficiently high, *C*_*X*_≈*C*_s_, and equation (2) can be simplified to


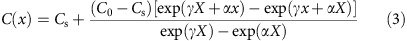


Equation (3) can be well fitted to the uranium concentration profiles observed on the modern seafloor^28^,^29^,^32^, if the proper *k*_U_ is applied.

For simplicity, we assume that the reaction for DIC in pore water is merely the degradation of organic matter. Thus, we describe the DIC concentration profile of pore water through the widely used 1G-Model for organic-matter degradation^48^:


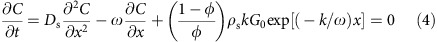


where *ρ*_s_ is the mean density of total sediment solids, *G*_0_ is the concentration of metabolizable organic carbon in the total solids at the sediment–seawater interface and *k* is the rate constant of organic carbon degradation, calculated through the empirical relationship *k=*0.057*ω*^1.94^ (ref. 49). The solution of equation (4) is


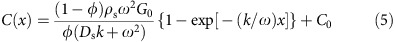


where *C*_0_ is the DIC concentration of bottom seawater. The profiles of the ^13^C and ^12^C concentrations are modelled, respectively, using equation (5) to obtain the pore water δ^13^C_DIC_ profile. The co-variation of δ^13^C_carb_ and U_carb_ can be modelled through the combination of equations (3) and (5). The parameters of the models are listed in Supplementary Table 6.

## Additional information

**How to cite this article:** Zhao, M.-Y. *et al.* Seeking a geochemical identifier for authigenic carbonate. *Nat. Commun.* 7:10885 doi: 10.1038/ncomms10885 (2016).

## Supplementary Material

Supplementary Information Supplementary Figures 1-7, Supplementary Tables 1-6, Supplementary Note 1 and Supplementary References.
