# Supplementary material for: Seeking a geochemical identifier for authigenic carbonate
Source: Nat Commun. 2016 Mar 7;7:10885. doi: 10.1038/ncomms10885 (PMC4786675; doi:10.1038/ncomms10885)
Supplement: Supplementary Information — Supplementary Figures 1-7, Supplementary Tables 1-6, Supplementary Note 1 and Supplementary References. [file ncomms10885-s1.pdf]

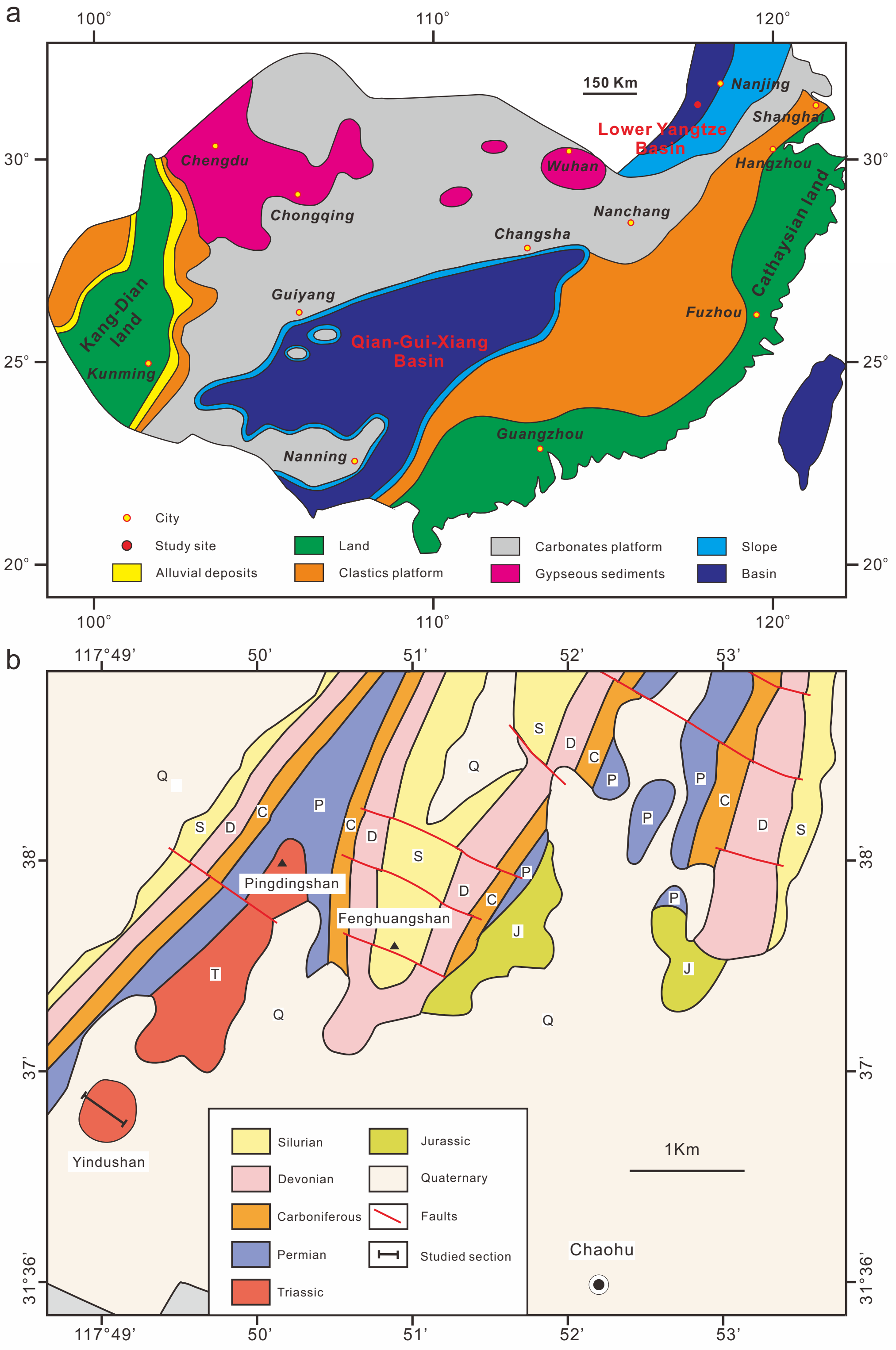

Supplementary Figure 1. Geological setting of the study area. a, Paleogeographical map of South China in the Olenekian, derived from ref. 1. **b,** Geological map of the study area in Chaohu City, Anhui Province, South China. The position of the studied Yindushan section is shown.

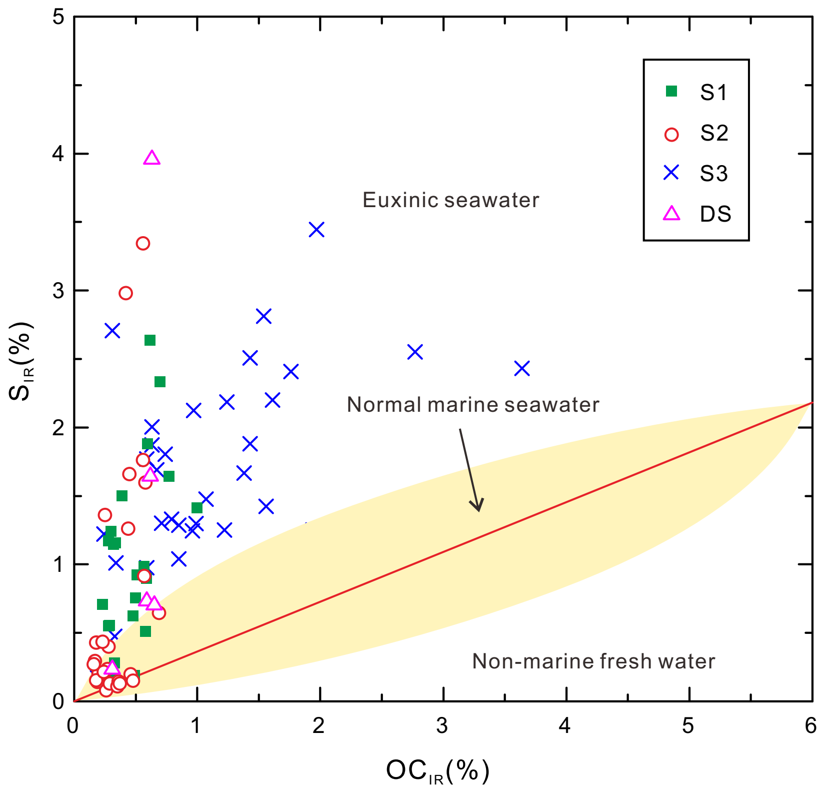

Supplementary Figure 2. Cross plot of S and OC contents for insoluble residues from the three intervals (S1, S2, S3). Dolomitic shale (DS) comes from S1. The yellow shadow denotes the region of normal marine sediments, derived from ref. 2. The red line denotes the mean correlation line for normal marine sediments, with a slope of 2.8.

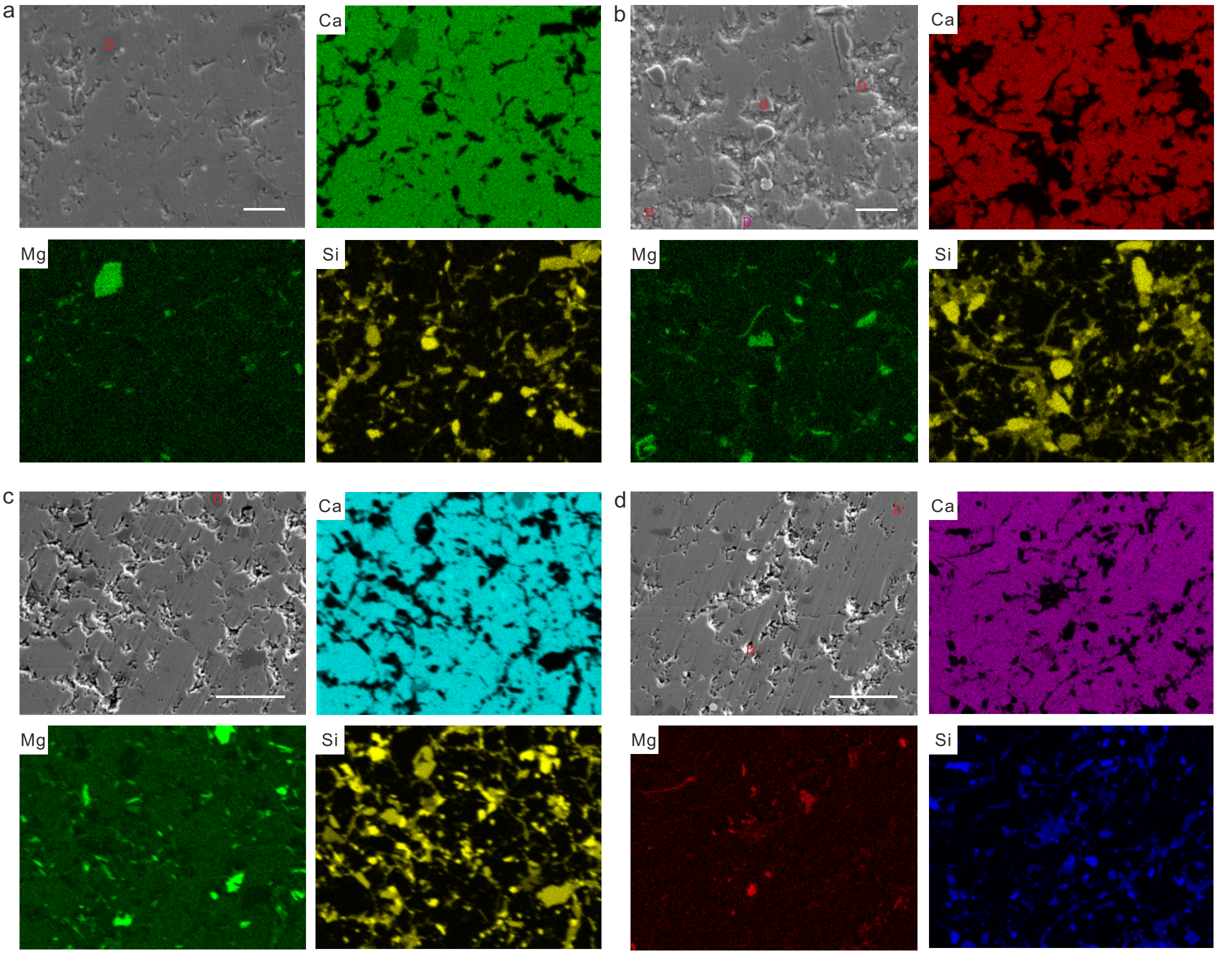

Supplementary Figure 3. Secondary electron images and results of energy spectrum analyses. a, core region in a limestone lense (Fig. 2a). **b,** equigranular microcrystalline region of a cement fringe (Fig. 2a). **c,** core region in a limestone lense (Fig. 2b). **d**, isopachous fibrous calcite (Fig. 2b). "D" denotes the dolomite. Other than sparse dolomite grains, the other regions with high Ca concentrations consist of calcite. The length of scale bars is 20 μm.

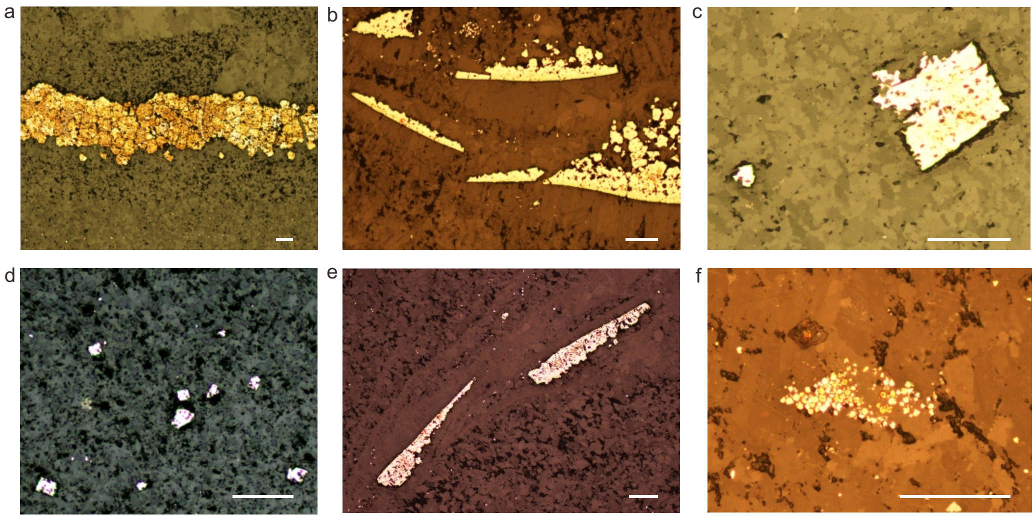

Supplementary Figure 4. The morphology of pyrite in the S1 of the Yindushan profile. a, a pyrite band in a cement fringe that was formed pervasively (Fig. 2a). **b,** pyrite assemblages in the isopachous fibrous calcite (Fig. 2b). **c,** a hypautomorphic pyrite in a core (Fig. 2a). **d,** xenomorphic pyrites in a central layer (Fig. 2c). **e,** pyrite assemblages in a core (Fig. 2b). **f,** framboidal pyrites in the isopachous fibrous calcite (Fig. 2b). Reflected light. The length of scale bars is 0.1 mm.

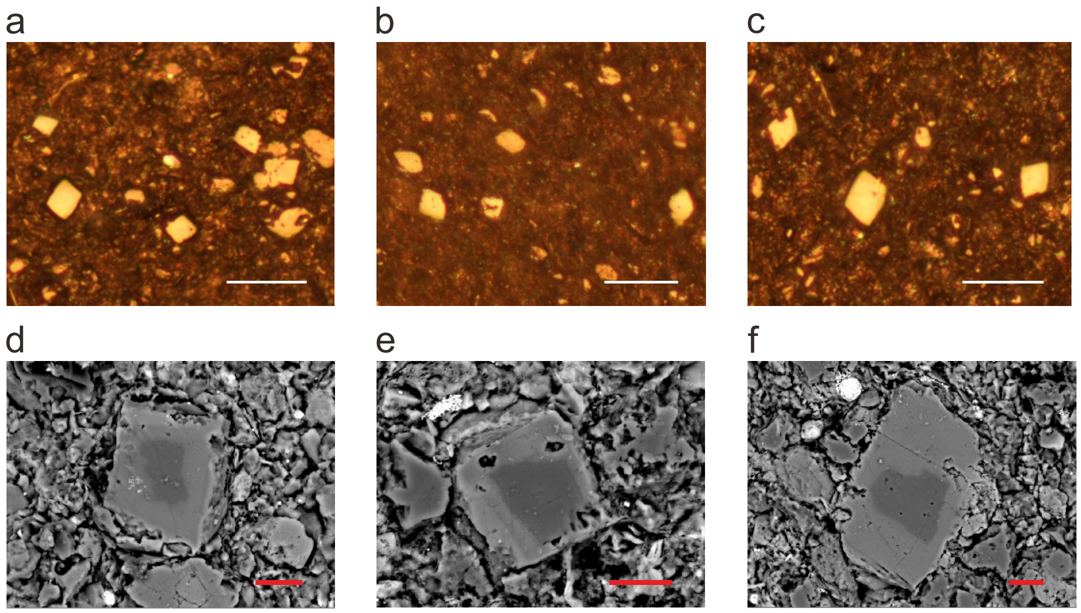

Supplementary Figure 5. Scattered ferroan dolomite rhombs in the dolomitic shale of S1. a-c, microscope observation in reflected light, showing the clean crystal faces of dolomite. **d-f,** backscattered electron (BSE) images, showing the iron-poor cores and iron-rich rims of dolomite. The lengths of scale bars are 50 μm in **a-c** and 10 μm in **d-f**, respectively.

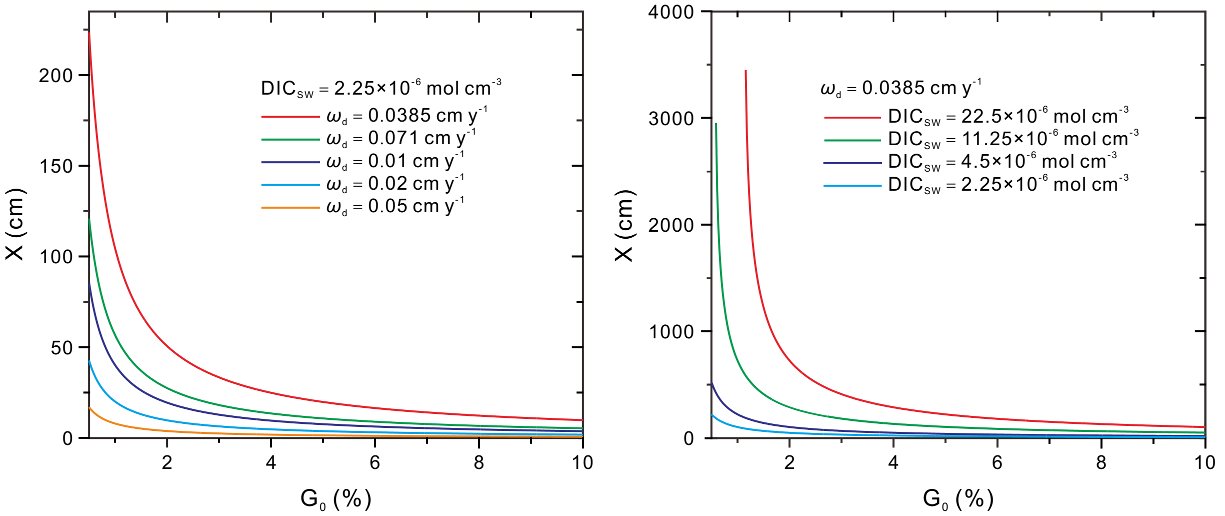

Supplementary Figure 6. Modelled maximum depth for the formation of the limestone lenses. The sample used in the model is corresponding to line b in Fig. 3. See the method part for the model construction.

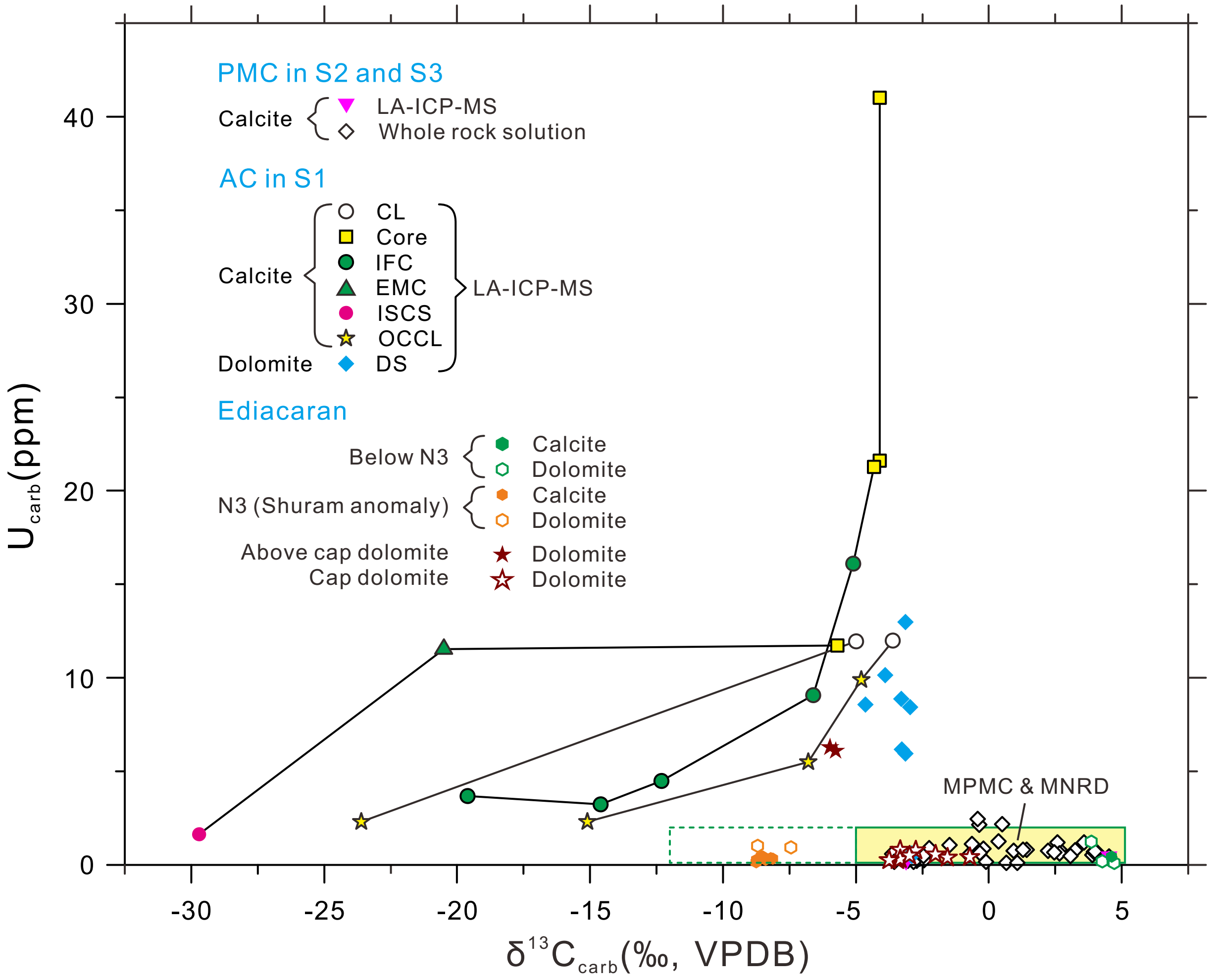

Supplementary Figure 7. A comparison of $\delta^{13}C_{carb}$ and U_{carb} results between the Ediacaran (Doushantuo Formation) and the Early Triassic (Yindushan section). See the legends of Figures 2 and 3 for the abbreviations. The dotted line outlines a guess on the region of ^{13}C-depleted primary marine carbonates ($\delta^{13}C < -5‰$).

Supplementary Table 1. $\delta^{13}C_{carb}$ and $\delta^{18}O_{carb}$ for the whole rocks and sub-samples and $\delta^{13}C_{org}$ for the insoluble residues in the Yindushan profile

Sample No.	Form	Height (m)	sub-samples	$\delta^{13}C_{carb}$ (VPDB, ‰)	$\delta^{18}O_{carb}$ (VPDB, ‰)	$\delta^{13}C_{org}$ (VPDB, ‰)
11CH701	layer	0		-3.14	-7.24	-26.7
			central layer	-2.21	-6.97	
			central layer	-2.08	-6.57	
			central layer	-1.92	-6.32	
			central layer	-1.96	-6.42	
			central layer	-3.94	-6.07	
			central layer	-4.53	-6.07	
			central layer	-3.78	-5.94	
			central layer	-4.19	-6.12	
			central layer	-4.96	-6.24	
			central layer	-5.33	-6.27	
			central layer	-4.50	-6.17	
11CH702	layer	0.4		-2.71	-6.66	-27.0
			cement fringe	-3.72	-7.20	
			cement fringe	-2.96	-7.00	
			cement fringe	-2.62	-7.63	
			cement fringe	-2.17	-6.57	
			central layer	-2.03	-6.50	
			central layer	-2.23	-7.02	
			central layer	-4.06	-6.82	
			central layer	-2.18	-6.46	
11CH703	layer	0.9		-4.39	-6.46	-24.2
			central layer	-5.74	-6.32	
			central layer	-3.84	-6.25	
			central layer	-5.36	-6.35	
			central layer	-3.94	-6.07	
			central layer	-3.61	-6.25	
			central layer	-3.86	-6.10	
			central layer	-4.54	-6.14	
			central layer	-3.94	-6.06	
			central layer	-3.44	-5.97	
			central layer	-3.47	-5.91	
11CH704	layer	1.3		-5.42	-6.75	-25.7
			central layer	-5.29	-6.21	
			central layer	-3.80	-7.54	
			central layer	-4.29	-8.46	
			central layer	-3.35	-8.00	
			central layer	-3.36	-7.05	
			central layer	-13.20	-6.79	
			central layer	-5.37	-8.10	
			central layer	-2.22	-6.73	

Supplementary Table 1 (continued)

Sample No.	Form	Height (m)	sub-samples	$\delta^{13}C_{carb}$ (VPDB, ‰)	$\delta^{18}O_{carb}$ (VPDB, ‰)	$\delta^{13}C_{org}$ (VPDB, ‰)
11CH705	layer	2.7		-3.44	-7.42	-26.0
			central layer	-2.75	-7.57	
			central layer	-3.55	-7.00	
			central layer	-3.66	-7.01	
			central layer	-3.39	-7.10	
			central layer	-2.89	-7.27	
11CH706	layer	3.1		-3.28	-7.02	-25.1
			central layer	-3.35	-8.03	
			central layer	-2.52	-6.11	
			central layer	-2.29	-6.49	
			central layer	-4.02	-6.17	
			central layer	-3.81	-7.90	
11CH707	layer	3.3		-4.38	-6.21	-26.8
			central layer	-2.69	-6.55	
			central layer	-3.01	-6.28	
			central layer	-3.58	-6.29	
			central layer	-4.52	-6.11	
			central layer	-4.12	-6.07	
			central layer	-4.46	-5.93	
			central layer	-4.39	-6.20	
			central layer	-4.25	-6.28	
11CH708	layer	3.7		-4.08	-6.33	-24.4
			central layer	-2.88	-7.13	
			central layer	-2.39	-6.46	
			central layer	-4.54	-6.28	
11CH709	layer	3.8				
			dolomitic shale	-2.91	-7.18	
			dolomitic shale	-3.35	-7.19	
			dolomitic shale	-2.86	-6.53	
			dolomitic shale	-4.49	-6.94	
11CH710	layer	3.9		-2.51	-6.98	-24.2
11CH711	layer	5.4		-3.77	-6.84	-24.5
			central layer	-4.10	-6.54	
			central layer	-4.01	-6.45	
			central layer	-3.19	-6.76	
11CH712	layer	6				
			dolomitic shale	-4.65	-6.46	
			dolomitic shale	-3.13	-6.90	
			dolomitic shale	-3.62	-6.36	
			dolomitic shale	-3.12	-6.77	
			dolomitic shale	-3.02	-7.06	

Supplementary Table 1 (continued)

Sample No.	Form	Height (m)	sub-samples	$\delta^{13}C_{carb}$ (VPDB, ‰)	$\delta^{18}O_{carb}$ (VPDB, ‰)	$\delta^{13}C_{org}$ (VPDB, ‰)
11CH713	lense	6.1		-6.40	-6.96	-27.0
			cement fringe	-20.48	-6.49	
			cement fringe	-19.96	-6.59	
			cement fringe	-19.15	-6.60	
			cement fringe	-12.85	-6.38	
			cement fringe	-5.87	-6.17	
			cement fringe	-4.32	-6.12	
			core	-3.85	-6.14	
			cement fringe	-14.16	-6.34	
			cement fringe	-7.47	-6.11	
			cement fringe	-5.45	-6.10	
			cement fringe	-4.15	-6.16	
			core	-3.86	-6.34	
11CH714	layer	7.1		-3.67	-8.13	-24.8
			cement fringe (OCCL)	-15.10	-6.83	
			cement fringe (OCCL)	-6.80	-6.34	
			cement fringe (OCCL)	-4.82	-6.43	
			central layer	-3.59	-8.32	
			central layer	-3.05	-9.07	
			cement fringe (OCCL)	-3.39	-8.12	
11CH715	layer	7.5				
			dolomitic shale	-3.26	-7.98	
			dolomitic shale	-2.96	-7.77	
			dolomitic shale	-3.27	-7.48	
			dolomitic shale	-5.16	-8.18	
			dolomitic shale	-2.94	-7.64	
			dolomitic shale	-5.23	-7.26	
11CH716	layer	8.4		-6.05	-7.49	-24.1
			central layer	-5.46	-7.64	
			central layer	-5.14	-7.97	
			central layer	-4.92	-8.23	
			central layer	-4.57	-8.19	
			central layer	-5.05	-7.55	
			central layer	-5.29	-6.97	
			central layer	-7.78	-6.59	
			central layer	-7.25	-6.58	
			central layer	-5.81	-7.38	
			central layer	-5.39	-7.97	
			central layer	-5.13	-8.13	
			central layer	-6.02	-6.72	
			central layer	-5.29	-7.01	
			central layer	-5.31	-7.80	
			central layer	-5.27	-8.20	

Supplementary Table 1 (continued)

Sample No.	Form	Height (m)	sub-samples	$\delta^{13}C_{carb}$ (VPDB, ‰)	$\delta^{18}O_{carb}$ (VPDB, ‰)	$\delta^{13}C_{org}$ (VPDB, ‰)
11CH717	lense	8.8		-7.41	-7.21	-26.6
			cement fringe	-23.40	-6.97	
			cement fringe	-23.57	-6.97	
			cement fringe	-23.42	-6.74	
			cement fringe	-23.55	-6.72	
			core	-6.84	-6.42	
			core	-5.44	-6.92	
			core	-5.73	-6.78	
			core	-6.11	-6.77	
			core	-5.68	-7.10	
			core	-5.73	-6.88	
			core	-5.87	-6.89	
			core	-6.36	-6.81	
			core	-7.20	-6.60	
			cement fringe (EMC)	-19.10	-6.89	
			cement fringe (EMC)	-19.42	-7.42	
			cement fringe (EMC)	-20.51	-7.36	
			cement fringe (EMC)	-19.17	-7.39	
			cement fringe (ISCS)	-28.76	-6.86	
			cement fringe (ISCS)	-29.54	-6.78	
			cement fringe (ISCS)	-29.67	-6.52	
11CH718	layer	9.8		-7.60	-7.17	-23.7
			central layer	-6.22	-6.79	
			central layer	-12.77	-6.70	
			central layer	-8.03	-6.44	
11CH719	lense	10.8		-3.71	-7.49	-26.8
11CH720	layer	11.8		-6.06	-7.22	-25.2
			central layer	-10.54	-6.72	
			central layer	-6.39	-6.71	
11CH721	layer	12.3				
			dolomitic shale	-4.97	-7.61	
			dolomitic shale	-3.13	-7.91	
			dolomitic shale	-3.90	-6.89	
			dolomitic shale	-3.53	-7.55	
			dolomitic shale	-5.17	-7.49	
			dolomitic shale	-7.38	-6.94	
11CH722	layer	13		-6.00	-6.68	-25.7
			cement fringe	-3.83	-7.32	
			cement fringe	-5.25	-7.10	
			cement fringe	-3.68	-6.90	
			cement fringe	-3.37	-6.94	
			cement fringe	-4.96	-6.68	
			cement fringe	-8.97	-6.20	

Supplementary Table 1 (continued)

Sample No.	Form	Height (m)	sub-samples	$\delta^{13}C_{carb}$ (VPDB, ‰)	$\delta^{18}O_{carb}$ (VPDB, ‰)	$\delta^{13}C_{org}$ (VPDB, ‰)
			cement fringe	-7.72	-6.46	
			cement fringe	-6.15	-6.14	
			central layer	-2.99	-6.87	
			central layer	-3.01	-6.93	
			central layer	-2.94	-6.78	
			central layer	-2.87	-6.91	
			central layer	-2.92	-6.66	
			central layer	-2.91	-6.79	
			central layer	-3.14	-6.59	
			central layer	-3.08	-6.57	
			central layer	-3.09	-6.61	
11CH723	layer	13.6		-7.05	-6.83	-24.9
			cement fringe (OCCL)	-19.88	-5.97	
			cement fringe (OCCL)	-21.52	-5.98	
			central layer	-6.32	-6.29	
			central layer	-4.85	-6.05	
			cement fringe (OCCL)	-23.63	-6.22	
			cement fringe (OCCL)	-21.19	-6.58	
			central layer	-6.20	-6.34	
			central layer	-4.97	-6.20	
11CH724	layer	14.5				
			dolomitic shale	-3.93	-7.64	
			dolomitic shale	-3.29	-7.44	
			dolomitic shale	-3.45	-7.49	
			dolomitic shale	-4.40	-7.68	
			dolomitic shale	-3.51	-7.51	
11CH725	lense	14.5		-7.87	-6.65	-27.5
			cement fringe (IFC)	-19.62	-5.99	
			cement fringe (IFC)	-14.63	-6.68	
			cement fringe (IFC)	-12.30	-6.57	
			cement fringe (IFC)	-6.58	-6.50	
			cement fringe (IFC)	-5.05	-6.43	
			core	-4.31	-6.30	
			core	-4.10	-6.24	
			core	-4.06	-6.20	
			core	-4.18	-6.30	
			core	-4.37	-6.35	
			cement fringe (IFC)	-4.89	-6.42	
			cement fringe (IFC)	-7.04	-6.69	
			cement fringe (IFC)	-9.89	-7.01	
			cement fringe (IFC)	-12.26	-6.96	
			cement fringe (IFC)	-16.22	-6.48	

Supplementary Table 1 (continued)

Sample No.	Form	Height (m)	sub-samples	$\delta^{13}C_{carb}$ (VPDB, ‰)	$\delta^{18}O_{carb}$ (VPDB, ‰)	$\delta^{13}C_{org}$ (VPDB, ‰)
			cement fringe (IFC)	-17.26	-6.01	
11CH726	lense	15.1		-4.50	-6.42	
			cement fringe	-6.99	-6.77	
			core	-4.75	-6.34	
			core	-4.23	-6.24	
			core	-4.16	-6.17	
			core	-3.99	-6.35	
			core	-4.00	-6.30	
			cement fringe	-5.03	-7.15	
11CH727	lense	15.9		-4.06	-6.77	
11CH728	layer	16.5		-3.93	-6.75	
			central layer	-3.88	-6.55	
			central layer	-3.66	-6.63	
			central layer	-3.49	-6.44	
			central layer	-4.34	-6.53	
			central layer	-5.04	-6.65	
			central layer	-3.93	-7.36	
			central layer	-4.28	-6.86	
			central layer	-4.17	-6.56	
			central layer	-3.94	-6.66	
			central layer	-4.10	-6.89	
11CH729	layer	16.9		-2.87	-6.84	
			central layer	-3.12	-6.48	
			central layer	-2.53	-6.95	
11CH730	layer	17.7		-2.73	-7.11	
11CH731	layer	18.8		-3.58	-6.63	
			central layer	-3.99	-7.38	
11CH732	layer	20.1		-3.63	-6.55	
11CH733	layer	20.6		-3.20	-6.45	
			central layer	-3.34	-6.21	
			central layer	-3.42	-6.06	
11CH734	layer	21.6		-3.56	-6.28	
11CH735	layer	22.6		-3.21	-6.21	
			central layer	-3.71	-5.13	
11CH736	layer	23.3		-3.04	-6.33	
11CH737	layer	24.4		-2.86	-6.63	
			central layer	-3.06	-6.37	
			central layer	-2.64	-6.61	
11CH738	layer	24.9		-3.08	-6.67	
11CH739	layer	25.6		-2.97	-6.64	
			central layer	-2.87	-6.40	
11CH740	layer	26.5		-2.59	-6.75	

Supplementary Table 1 (continued)

Sample No.	Form	Height (m)	sub-samples	$\delta^{13}C_{carb}$ (VPDB, ‰)	$\delta^{18}O_{carb}$ (VPDB, ‰)	$\delta^{13}C_{org}$ (VPDB, ‰)
11CH741	layer	27.5		-2.61	-6.66	
11CH742	layer	28.5		-2.70	-6.56	
11CH743	layer	29.6		-2.60	-6.37	
			central layer	-2.88	-6.70	
			central layer	-2.64	-6.23	
11CH744	layer	30.2		-2.54	-6.58	
11CH745	layer	30.8		-2.69	-6.71	
			central layer	-2.69	-6.40	
11CH746	layer	31.2		-2.43	-6.50	
11CH747	layer	31.75		-2.49	-6.74	
			central layer	-2.56	-6.49	
			central layer	-2.58	-6.57	
11CH748	layer	32.15		-2.83	-6.74	
11CH749	layer	32.65		-2.63	-6.36	
			central layer	-2.95	-5.79	
			central layer	-3.06	-5.78	
11CH750	layer	33.05		-2.24	-6.65	
11CH751	layer	33.85		-1.71	-6.70	
			central layer	-1.56	-6.57	
			central layer	-1.67	-6.50	
11CH752	layer	34.45		-2.46	-5.56	
11CH753	layer	35.15		-0.84	-5.87	
			central layer	-0.95	-5.49	
11CH754	layer	35.65		-0.75	-5.82	
11CH769	layer	37.15		1.21	-6.29	
11CH791	layer	41.35		2.23	-5.59	
11CH792	layer	42.55		2.65	-6.42	
11CH793	layer	43.55		2.89	-6.05	
11CH794	layer	44.55		3.88	-6.15	
11CH795	layer	46.55		4.33	-5.73	
11CH796	layer	48.45		4.52	-6.10	
11CH797	layer	50.15		4.17	-6.11	
11CH798	layer	51.65		3.95	-5.65	
11CH799	layer	53.15		3.95	-5.70	
11CH800	layer	54.35		3.24	-6.30	
11CH801	layer	55.85		3.80	-5.66	
11CH802	layer	57.25		3.58	-6.27	
11CH803	layer	59.25		2.98	-6.51	
11CH804	layer	60.75		2.58	-6.73	
11CH805	layer	62.25		2.28	-7.08	
11CH806	layer	63.75		2.66	-6.85	
11CH807	layer	65.75		3.04	-7.26	

Supplementary Table 1 (continued)

Sample No.	Form	Height (m)	sub-samples	$\delta^{13}C_{carb}$ (VPDB, ‰)	$\delta^{18}O_{carb}$ (VPDB, ‰)	$\delta^{13}C_{org}$ (VPDB, ‰)
11CH808	layer	67.25		3.06	-7.16	
11CH809	layer	68.75		2.39	-6.21	
11CH810	layer	70.75		4.05	-5.83	
11CH811	layer	72.25		3.88	-5.99	
11CH812	layer	74.25		3.98	-6.11	
11CH813	layer	78.25		2.90	-6.60	
11CH814	layer	80.25		2.23	-6.81	
11CH815	layer	81.75		0.88	-5.26	
11CH816	layer	83.75		-0.01	-5.93	
11CH817	layer	85.75		0.09	-6.07	
11CH818	layer	87.25		0.37	-5.90	
11CH819	layer	88.75		0.44	-5.77	
11CH820	layer	90.75		0.67	-5.72	
11CH821	layer	92.75		0.78	-5.77	
11CH822	layer	93.75		0.51	-6.66	
11CH823	layer	95.25		0.69	-6.13	
11CH824	layer	97.25		0.94	-6.34	
11CH825	layer	98.75		0.96	-6.31	
11CH826	layer	100.25		1.07	-6.20	
11CH827	layer	102.25		1.12	-6.11	
11CH828	layer	103.75		1.43	-5.73	
11CH829	layer	105.25		1.43	-6.08	
11CH830	layer	106.75		2.45	-5.57	
11CH831	layer	108.25		1.09	-6.24	
11CH832	layer	110.25		1.28	-6.30	
11CH833	layer	111.75		1.11	-5.79	
11CH834	layer	113.25		-0.11	-6.07	
11CH835	layer	114.75		-0.62	-5.72	
11CH836	layer	116.75		-0.36	-6.96	
11CH837	layer	117.75		-0.52	-6.67	
11CH838	layer	118.75		-0.43	-6.42	
11CH839	layer	119.75		-0.43	-6.48	
11CH840	layer	120.75		-0.20	-6.17	
11CH841	layer	121.75		-0.32	-6.29	
11CH842	layer	122.75		-0.64	-6.72	
11CH843	layer	123.75		-0.83	-6.46	
11CH844	layer	124.75		-1.48	-6.48	
11CH845	layer	125.85		-1.59	-6.34	
11CH846	layer	126.85		-0.33	-6.76	
11CH847	layer	127.85		-0.99	-7.44	

Supplementary Table 2. Insoluble residue (IR) contents, organic carbon and sulfur contents of insoluble residues (OC_{IR} and S_{IR}) and U, P and Al concentrations of carbonate in whole-rock samples of the Yindushan profile

Sample No.	IR (%)	OC_{IR} (%)	S_{IR} (%)	U_{carb} (ppm)	P_{carb} (ppm)	Al_{carb} (%)
11CH701	33.0	0.49	0.19	1.31	62	0.231
11CH702	27.1	0.60	1.88	9.28	79	0.093
11CH703	23.7	0.32	1.15	2.09	79	0.109
11CH704	4.8	1.00	1.41	6.57	104	0.047
11CH705	15.8	0.48	0.62	5.16	93	0.094
11CH706	15.8	0.33	0.28	1.67	85	0.091
11CH707	15.4	0.50	0.75	6.39	94	0.063
11CH708	23.1	0.23	0.71	0.63	101	0.129
11CH709	81.4	0.31	0.24			
11CH710	28.4	0.31	0.22	1.96	94	0.182
11CH711	18.9	0.29	0.56	1.92	76	0.108
11CH712	78.4	0.62	1.65			
11CH713	16.4	0.51	0.93	11.57	86	0.140
11CH714	20.4	0.28	0.55	7.21	101	0.145
11CH715	81.2	0.59	0.74			
11CH716	17.9	0.39	1.50			
11CH717	13.7	0.62	2.64	9.62	87	0.125
11CH718	19.6	0.30	1.24	3.06	99	0.112
11CH719	11.2	0.58	0.51	17.94	119	0.083
11CH720	16.5	0.34	1.16	3.92	77	0.097
11CH721	77.2	0.63	3.96			
11CH722	13.8	0.59	0.90	6.53	111	0.073
11CH723	20.0	0.28	1.17	6.91	171	0.121
11CH724	82.0	0.65	0.71			
11CH725	24.4	0.57	0.99	11.39	72	0.146
11CH726	15.2	0.77	1.64	14.99	124	0.091
11CH727	19.6	0.70	2.33			
11CH728	25.3	0.58	1.60	11.43	153	0.110
11CH729	6.6	0.57	0.92			
11CH730	5.6	0.69	0.65	1.71	36	0.049
11CH731	17.2	0.27	0.24			
11CH732	21.9	0.20	0.20	0.58	31	0.106
11CH733	32.2	0.17	0.30			
11CH734	16.8	0.26	0.08	0.18	29	0.106
11CH735	39.4	0.18	0.43			
11CH736	34.9	0.19	0.14	0.23	24	0.193
11CH737	39.1	0.18	0.16			
11CH738	36.8	0.16	0.27	0.25	34	0.203
11CH739	30.4	0.24	0.22			
11CH740	42.0	0.24	0.16	0.34	17	0.247
11CH741	35.2	0.46	0.20			
11CH742	30.6	0.29	0.13	0.23	12	0.171

Supplementary Table 2 (continued)

Sample No.	IR (%)	OC_{IR} (%)	S_{IR} (%)	U_{carb} (ppm)	P_{carb} (ppm)	Al_{carb} (%)
11CH743	8.2	0.48	0.15			
11CH744	25.8	0.36	0.15	0.46	21	0.252
11CH745	29.3	0.35	0.11			
11CH746	41.4	0.28	0.40	0.25	20	0.241
11CH747	16.5	0.37	0.13			
11CH748	22.1	0.23	0.44	0.16	27	0.158
11CH749	22.7	0.25	1.36			
11CH750	17.0	0.56	1.76	0.88	40	0.097
11CH751	22.9	0.45	1.66			
11CH752	6.2	0.44	1.26	0.47	40	0.051
11CH753	20.9	0.42	2.98			
11CH754	15.3	0.56	3.34	0.52	33	0.119
11CH791	5.2	0.63	1.87			
11CH792	3.1	0.90	0.29	0.82	24	0.056
11CH793	3.4	0.59	0.98			
11CH794	1.1	2.77	2.55	0.53	17	0.029
11CH795	2.2	0.99	1.30			
11CH796	1.8	1.94	1.25	0.46	20	0.031
11CH797	2.8	0.79	1.33			
11CH798	2.9	1.36	0.48	0.70	25	0.056
11CH799	2.7	1.97	3.45			
11CH800	19.7	0.97	2.13	0.77	40	0.086
11CH801	12.5	0.63	2.01			
11CH802	13.6	0.59	1.77	1.20	39	0.114
11CH803	3.3	0.84	0.69			
11CH804	2.1	1.61	2.20	1.20	27	0.035
11CH805	1.6	3.68				
11CH806	1.2	1.59	0.56	0.61	19	0.024
11CH807	1.5	1.22	0.39			
11CH808	2.0	1.31	0.39	0.48	17	0.029
11CH809	5.9	0.38	0.22			
11CH810	1.7	1.76	2.41	0.69	14	0.037
11CH811	1.9	2.03	0.48			
11CH812	1.5	3.64	2.43	0.72	12	0.032
11CH813	16.4	0.34	1.01			
11CH814	3.5	0.86	0.25	0.76	12	0.051
11CH815	38.0	0.31	2.71			
11CH816	40.2	0.19	0.18	0.34	44	0.161
11CH817	20.0	0.18	0.22			
11CH818	2.9	0.69	0.56	1.25	13	0.029
11CH819	3.3	0.61				
11CH820	19.4	0.22	0.17	0.12	41	0.091
11CH821	2.7	0.55	0.35			

Supplementary Table 2 (continued)

Sample No.	IR (%)	OC_{IR} (%)	S_{IR} (%)	U_{carb} (ppm)	P_{carb} (ppm)	Al_{carb} (%)
11CH822	1.7	1.38	1.67	2.17	14	0.024
11CH823	18.2	0.24	1.22			
11CH824	2.4	1.52	0.47	0.74	10	0.026
11CH825	2.2	1.02	0.31			
11CH826	11.9	0.33	0.48	0.12	17	0.080
11CH827	4.4	1.24	2.19			
11CH828	4.3	0.85	1.04	0.80	17	0.047
11CH829	2.9	1.00	0.61			
11CH830	7.9	0.60	0.56	0.67	24	0.062
11CH831	25.4	0.21	0.19			
11CH832	2.9	0.96	1.25	0.81	24	0.028
11CH833	3.1	0.85	1.29			
11CH834	28.6	0.27	0.19	0.17	53	0.147
11CH835	37.8	0.28	0.16			
11CH836	9.0	0.46	0.33	2.14	54	0.224
11CH837	7.5	0.67	1.69			
11CH838	9.5	0.71	1.30	2.44	27	0.066
11CH839	7.9	0.74	1.81			
11CH840	4.5	1.54	2.81	0.86	25	0.042
11CH841	4.7	1.43	2.51			
11CH842	7.8	1.56	1.42	1.10	29	0.076
11CH843	3.9	1.43	1.88			
11CH844	14.9	1.31	0.97	1.05	53	0.077
11CH845	15.7	1.07	1.48			
11CH846	7.4	1.22	1.25	0.63	26	0.037
11CH847	4.2	1.54	0.95			

Supplementary Table 3. Microscale U concentrations of carbonate analyzed using LA-ICP-MS and the corresponding $\delta^{13}C_{carb}$ values of the Yindushan profile.

Sample No.	Sub-samples	$\delta^{13}C_{carb}$ (VPDB, ‰)	U_{carb} (ppm)
11CH717	Irregular-stacked calcite spar	-29.67	1.65
11CH717	Equigranular microcrystalline calcite	-20.51	11.53
11CH717	Core	-5.73	11.73
11CH725	Isopachous fibrous calcite	-19.62	3.66
11CH725	Isopachous fibrous calcite	-14.63	3.21
11CH725	Isopachous fibrous calcite	-12.30	4.49
11CH725	Isopachous fibrous calcite	-6.58	9.06
11CH725	Isopachous fibrous calcite	-5.05	16.08
11CH725	Core	-4.31	21.26
11CH725	Core	-4.10	21.59
11CH725	Core	-4.06	40.99
11CH714	Outward-coursing calcite laminaes	-15.10	2.32
11CH714	Outward-coursing calcite laminaes	-6.80	5.51
11CH714	Outward-coursing calcite laminaes	-4.82	9.89
11CH714	Central layer	-3.59	11.99
11CH723	Outward-coursing calcite laminaes	-23.63	2.32
11CH723	Central layer	-4.97	11.94
11CH709-1	Dolomitic shale	-2.91	0.54
11CH712-1	Dolomitic shale	-4.65	8.54
11CH712-2	Dolomitic shale	-3.13	5.92
11CH715-1	Dolomitic shale	-2.96	8.42
11CH715-2	Dolomitic shale	-3.27	6.17
11CH721-1	Dolomitic shale	-3.13	12.95
11CH721-2	Dolomitic shale	-3.90	10.12
11CH724-1	Dolomitic shale	-3.29	8.86
11CH738	Primary marine carbonate	-3.08	0.21
11CH796	Primary marine carbonate	4.52	0.49

Supplementary Table 4. $\delta^{13}C_{carb}$, $\delta^{18}O_{carb}$ and U_{carb} results of microcrystalline dolomite in Triassic and Ediacaran periods.

Sample No.	Dolomite Type	Age	Location	Formation	Rock	$\delta^{13}C_{carb}$ (VPDB, ‰)	$\delta^{18}O_{carb}$ (VPDB, ‰)	U_{carb} (ppm)	Method
12CH01	Non-authigenic	Triassic, Anisian	Chaohu, Anhui, China	Dongmaanshan	Evaporite collapse breccias	1.50	-2.79	0.62	Solution
12CH05	Non-authigenic	Triassic, Anisian	Chaohu, Anhui, China	Dongmaanshan	Evaporite collapse breccias	1.68	-4.05	1.21	Solution
11YC2-1	Non-authigenic	Ediacaran	Yichang, Hubei, China	Doushantuo	Cap dolomite	-1.56	-5.48	0.37	LA-ICP-MS
11YC2-3	Non-authigenic	Ediacaran	Yichang, Hubei, China	Doushantuo	Cap dolomite	-3.42	-7.72	0.24	LA-ICP-MS
11YC3-1	Non-authigenic	Ediacaran	Yichang, Hubei, China	Doushantuo	Cap dolomite	-2.00	-6.95	0.52	LA-ICP-MS
10WH01	Non-authigenic	Ediacaran	Wuhe, Guizhou, China	Doushantuo	Cap dolomite	-2.74	-6.49	0.76	LA-ICP-MS
10WH03	Non-authigenic	Ediacaran	Wuhe, Guizhou, China	Doushantuo	Cap dolomite	-3.34	-6.45	0.79	LA-ICP-MS
10WH05	Non-authigenic	Ediacaran	Wuhe, Guizhou, China	Doushantuo	Cap dolomite	-3.34	-9.59	0.28	LA-ICP-MS
10WH07	Non-authigenic	Ediacaran	Wuhe, Guizhou, China	Doushantuo	Cap dolomite	-2.98	-6.81	0.48	LA-ICP-MS
10WH10	Non-authigenic	Ediacaran	Wuhe, Guizhou, China	Doushantuo	Cap dolomite	-3.74	-6.87	0.22	LA-ICP-MS
10WH13	Non-authigenic	Ediacaran	Wuhe, Guizhou, China	Doushantuo	Cap dolomite	-0.72	-3.01	0.40	LA-ICP-MS
10WH14	Authigenic	Ediacaran	Wuhe, Guizhou, China	Doushantuo	Dolomite layer in black shale	-5.97	-8.73	6.26	LA-ICP-MS
10WH15	Authigenic	Ediacaran	Wuhe, Guizhou, China	Doushantuo	Dolomite layer in black shale	-5.76	-8.17	6.06	LA-ICP-MS

Supplementary Table 5. $\delta^{13}C_{carb}$, $\delta^{18}O_{carb}$, U_{carb}, P_{carb} and Al results of the upper part of Ediacaran Doushantuo Formation at the Jiulongwan section.

Interval	Sample No.	Mineral	$\delta^{13}C_{carb}$ (VPDB, ‰)	$\delta^{18}O_{carb}$ (VPDB, ‰)	U_{carb} (ppm)	P_{carb} (ppm)	Al (%)	$U_{average}$ (ppm)	$P_{average}$ (ppm)
Below N3	12JLW-26	dolomite	4.28	-4.22	0.17	174	0.039	0.48 ± 0.52	149 ± 47
	12JLW-28	calcite	4.62	-8.73	0.41	78	0.009		
	12JLW-29	dolomite	4.73	-5.79	0.09	178	0.012		
	12JLW-30	dolomite	3.87	-7.99	1.23	164	0.011		
N3 (Shuram anomaly)	12JLW-40	calcite	-8.72	-9.38	0.25	76	0.017	0.43 ± 0.29	94 ± 54
	12JLW-44	calcite	-8.75	-9.14	0.11	56	0.018		
	12JLW-45	calcite	-8.63	-8.63	0.28	75	0.010		
	12JLW-47	calcite	-8.39	-8.44	0.25	100	0.023		
	12JLW-48	calcite	-8.49	-8.64	0.43	60	0.013		
	12JLW-49	calcite	-8.19	-8.30	0.35	47	0.008		
	12JLW-51	calcite	-8.12	-6.26	0.30	86	0.011		
	12JLW-52	calcite	-8.56	-7.47	0.44	59	0.011		
	12JLW-53	dolomite	-7.43	-2.22	0.91	165	0.017		
	12JLW-54	dolomite	-8.69	-1.53	1.00	214	0.017		

Note: U_{carb}, P_{carb} and Al were analyzed through the whole-rock solution method.

Supplementary Table 6. The parameters used in the model.

Parameter	Description	Value	Unit
ω_d	Sedimentation rate of compacted strata	0.00385-0.05 [a]	cm y^{-1}
ω	Sediment advection rate	$\omega_d/(1-\phi)$	cm y^{-1}
G_0	Concentration of metabolizable organic carbon in the total solids at the sediment-seawater interface	4.17-83.3×10^{-4} [b]	mol g^{-1}
k	Rate constant for organic carbon degradation	0.057×$\omega^{1.94}$ [c]	y^{-1}
k_U	First order remove rate constant for U	10^{-8}-10^{-6} [d]	s^{-1}
$\delta^{13}C_{org}$	δ^{13}C of metabolizable organic carbon	-27.5 [e]	‰
$\delta^{13}C_{SW}$	δ^{13}C of bottom seawater	-2.8 [f]	‰
ϕ	Porosity	75.6 [g]	%
ρ_s	Mean density of total sediment solids	2.6	g cm^{-3}
D_s	Sediment diffusion coefficient	$D_M\times\phi^2$	cm^2 s^{-1}
D_M	Free solution diffusion coefficients	11.8×10^{-6} (HCO_3^-, 25 °C) [h]	cm^2 s^{-1}
		4.26×10^{-6} (UO_2^{2+}, 25 °C) [i]	cm^2 s^{-1}
C_0	Concentrations of bottom seawater	2.25-22.5 (DIC) [j]	μmol cm^{-3}
		13 (U) [k]	nmol kg^{-1}

a. Estimated for the Smithian through a thickness of ~50 m and a duration of 0.7±0.6 My[3].

b. Equivalent to 0.5-10%.

c. Derived from ref. 4.

d. Estimated through applying the model to a few U concentration profiles in modern seafloor.

e. Equivalent to $\delta^{13}C_{org}$ value of the sample in the model.

f. Equivalent to average $\delta^{13}C_{carb}$ value of the primary marine carbonate in the Smithian.

g. Equivalent to carbonate volume percentage of the sample in the model with the assumption that the precipitation of authigenic calcite completely replaced pore space without overgrowth[5].

h. Derived from ref. 6.

i. Derived from ref. 6.

j. 1-10 times of modern value.

k. Modern value.

Supplementary Note 1. Geological setting

1. Early Triassic Yindushan section

The South China Block was located at a low latitude in the eastern edge of the Paleotethyan ocean during the Early Triassic[7]. It was composed of two lands (Cathaysian and Kang-Dian), two basins (Lower Yangtze and Qian-Gui-Xiang), several gypseous lagoons and a united platform filled with carbonates and terrigenous detritus (Supplementary Fig. 1). The study region was situated in the deep water part of the Lower Yangtze basin that was connected to the open ocean in the Early Triassic[1]. Since the South China Block and the North China Block were converging during the Early Triassic, strata of the Lower Yangtze basin in this period show a regressive trend. The studied Yindushan section crops out in the core of Yindushan-Pingdingshan syncline in vicinity of Chaohu city, Anhui province (Supplementary Fig. 1). So far, extensive investigations have been performed in the study region for carbon isotope stratigraphy, paleomagnetic stratigraphy, lithostratigraphy and palaeontology of the Early Triassic[8-11]. C-S relationship indicates that the adjacent West Pingdingshan Section was deposited in a euxinic environment[12].

2. Ediacaran Jiulongwan section

The Ediacaran Doushantuo Formation has a wide distribution in the west part of South China on a passive continental margin. The studied Jiulongwan section was formed in shallow water near or below wave base, and the evidence of subaerial exposure has not been observed[13,14]. Sedimentological evidences indicate that the studied member III containing the interval comparable with the Shuram anomaly (N3) represents a regressive sequence[13], with alternative limestone-dolostone “ribbon rocks”. The mineral compositions of the study samples were identified through XRD (Supplementary Table 5). The interval below Shuram anomaly is dominant by dolostone, while the N3 interval is dominant by limestone. Fe mineral species indicate that the carbonate rocks in the member III were deposited in a ferruginous environment[14]. The two dolomite samples in N3 of this study have $\delta^{18}O_{carb}$ values near to -2‰ (Supplementary Table 5), indicating the resistance of dolomite to meteoric and deep burial diagenesis. The similar $\delta^{13}C_{carb}$ values between calcite and dolomite indicate that the influence of meteoric and deep burial diagenesis on the $\delta^{13}C_{carb}$ of calcite may be minor. It was found that the $\delta^{34}S$ values of pyrite in N3 have a large offset (~30‰) with those of the co-existing sulfate[14], inconsistent with substantial diagenetic reduction of sulfate in a semi-closed system.

Supplementary References

1. Feng, Z. Z., Bao, Z. D. & Li, S. W. *Lithofacies Paleogeography of Middle and Lower Triassic of South China* (Pet. Indust. Press, Beijing, 1997) (in Chinese with English abstract).
2. Berner, R. A. & Raiswell, R. Burial of organic carbon and pyrite sulfur in sediments over Phanerozoic time: a new theory. *Geochim. Cosmochim. Acta* **47,** 855–862 (1983).
3. Galfetti, T. *et al.* Timing of the Early Triassic carbon cycle perturbations inferred from new U–Pb ages and ammonoid biochronozones. *Earth Planet. Sci. Lett.* **258,** 593–604 (2007).

4. Tromp, T. K., Van Cappellen, P. & Key, R. M. A global model for the early diagenesis of organic carbon and organic phosphorus in marine sediments. *Geochim. Cosmochim. Acta* **59,** 1259–1284 (1995).
5. Curtis, C. D., Coleman, M. L. & Love, L. G. Pore water evolution during sediment burial from isotopic and mineral chemistry of calcite, dolomite and siderite concretions. *Geochim. Cosmochim. Acta* **50,** 2321–2334 (1986).
6. Li, Y.-H. & Gregory, S. Diffusion of ions in sea water and in deep-sea sediments. *Geochim. Cosmochim. Acta* **38,** 703–714 (1974).
7. Luo, G. M. *et al.* Isotopic evidence for an anomalously low oceanic sulfate concentration following end-Permian mass extinction. *Earth Planet. Sci. Lett.* **300,** 101–111 (2010).
8. Sun, Z. *et al.* Magnetostratigraphy of the Lower Triassic beds from Chaohu (China) and its implications for the Induan-Olenekian stage boundary. *Earth Planet. Sci. Lett.* **279,** 350–361 (2009).
9. Tong, J., Qiu, H., Zhao, L. & Zuo, J. Lower Triassic inorganic carbon isotope excursion in Chaohu, Anhui Province, China. *J. China Univ. Geosci.* **13,** 98–106 (2002).
10. Tong, J., Zuo, J. & Chen, Z. Q. Early Triassic carbon isotope excursions from South China: proxies for devastation and restoration of marine ecosystems following the end-Permian mass extinction. *Geol. J.* **42,** 371–389 (2007).
11. Zhao, L. *et al.* Lower Triassic conodont sequence in Chaohu, Anhui Province, China and its global correlation. *Palaeogeog. Palaeoclimatol. Palaeoecol.* **252,** 24–38 (2007).
12. Zhao, M. Y. & Zheng, Y. F. The intensity of chemical weathering: Geochemical constraints from marine detrital sediments of Triassic age in South China. *Chem. Geol.* **391,** 111–122 (2015).
13. McFadden, K. A. *et al.* Pulsed oxidation and biological evolution in the Ediacaran Doushantuo Formation. *Proc. Nat. Acad. Sci. USA* **105,** 3197–3202 (2008).
14. Li, C. *et al.* A stratified redox model for the Ediacaran ocean. *Science* **328,** 80–83 (2010).
